# Supplementary material for: Phylogenomic Analyses of Three Distinct Lineages Uniting Staphylococcus cohnii and Staphylococcus urealyticus from Diverse Hosts
Source: Microorganisms. 2024 Jul 29;12(8):1549. doi: 10.3390/microorganisms12081549 (PMC11356006; doi:10.3390/microorganisms12081549)

Supplemental Figures

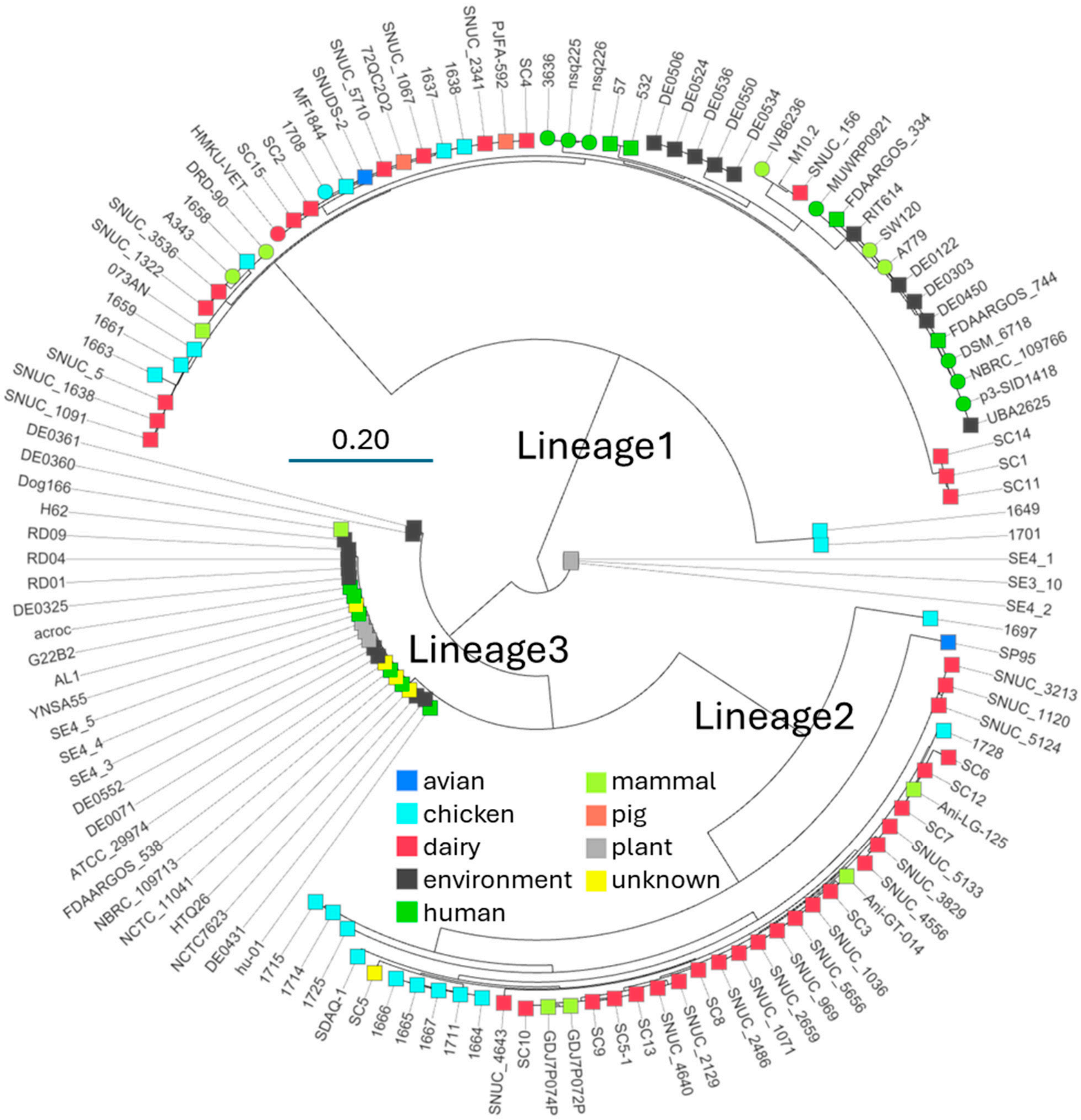

Figure S1. ParSNP phylogenomic tree of 126 genomes of isolates of *Staphylococcus cohnii* (squares) and *Staphylococcus urealyticus (circles)* with FDAARGOS_538 as reference, based on 82,870 core genome SNPs. Strain names are the leaves, with nodes colored according to host as determined from NCBI Biosample entries. The branches for the three lineages discussed in the text are indicated.

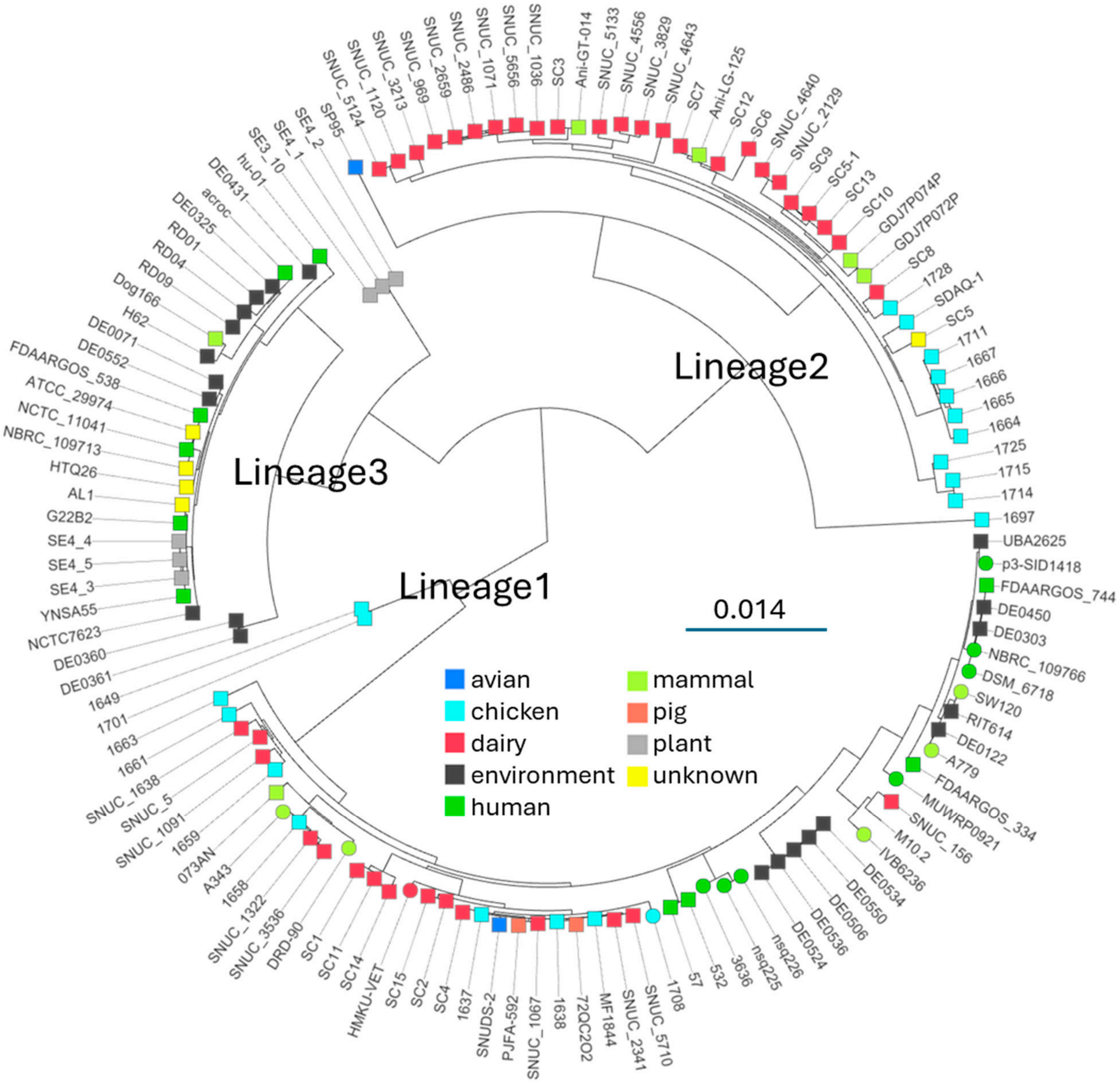

Figure S2. pyANI phylogenomic tree of 126 genomes of isolates of *Staphylococcus cohnii* (squares) and *Staphylococcus urealyticus (circles)*. Strain names are the leaves, with nodes colored according to host as determined from NCBI Biosample entries. The branches for the three lineages discussed in the text are indicated.

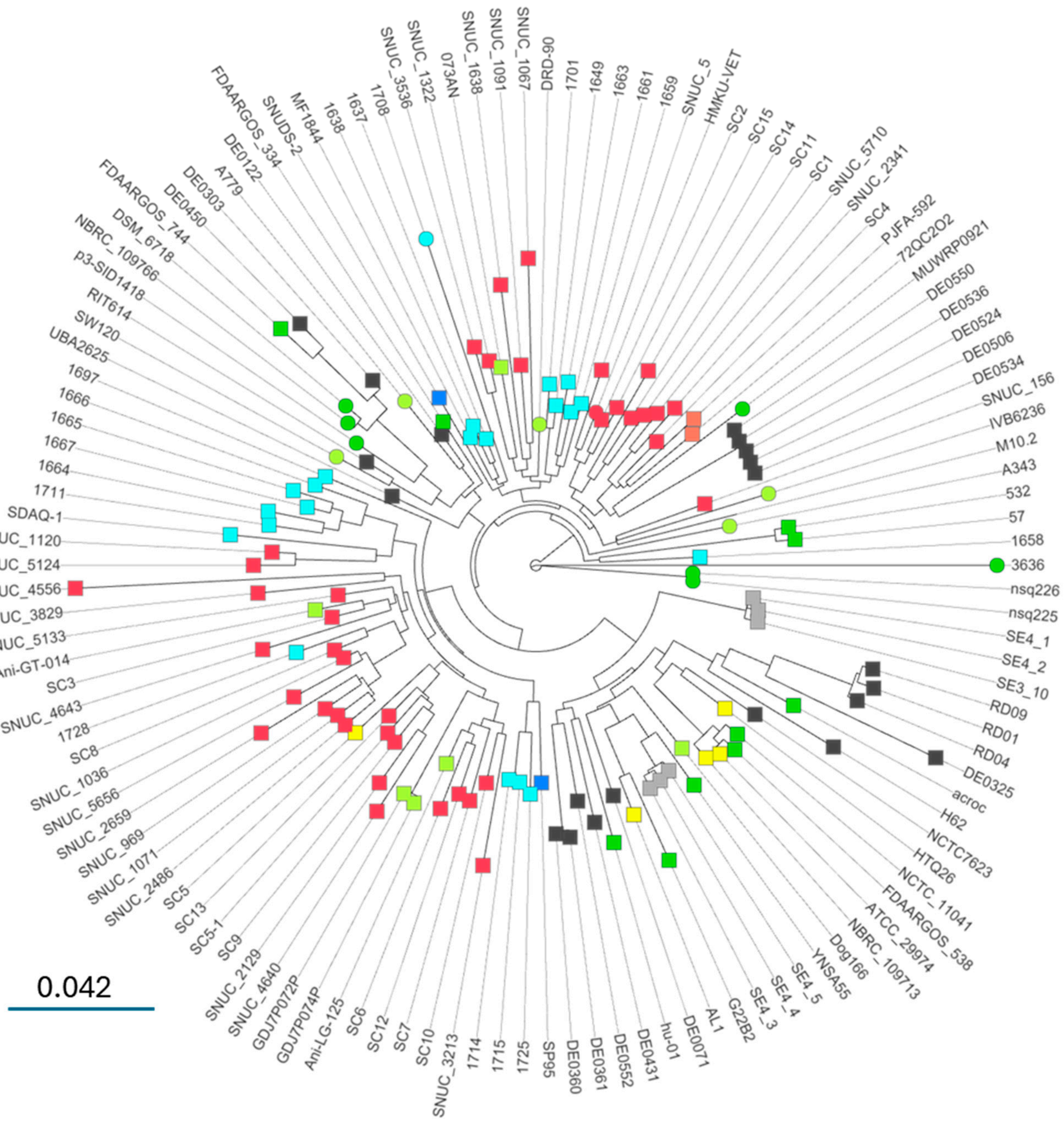

Figure S3. Percentage of Conserved Proteins phylogenomic tree of 126 genomes of isolates of *Staphylococcus cohnii* (squares) and *Staphylococcus urealyticus (circles)* isolates. Strain names are the leaves, with nodes colored according to host as determined from NCBI Biosample entries.

Figure S4. FastANI alignments from ProkSee server of representative complete genomes of *Staphylococcus nepalensis* and the three lineages of *Staphylococcus cohnii*. Genomes are L1: Lineage 1, FDAARGOS_334; L2: Lineage 2, SDAQ-1; L3: Lineage 3, FDAARGOS_538 reverse complement; and Sn: *S. nepalensis*, JS11. For the top panel the central genome is Lineage 1 compared to Lineage 3 above and Lineage 2 below. The other panels are each lineage compared to *S. nepalensis*. Approximate genomic distance is indicated at the bottom along with the heat map for the connecting lines.

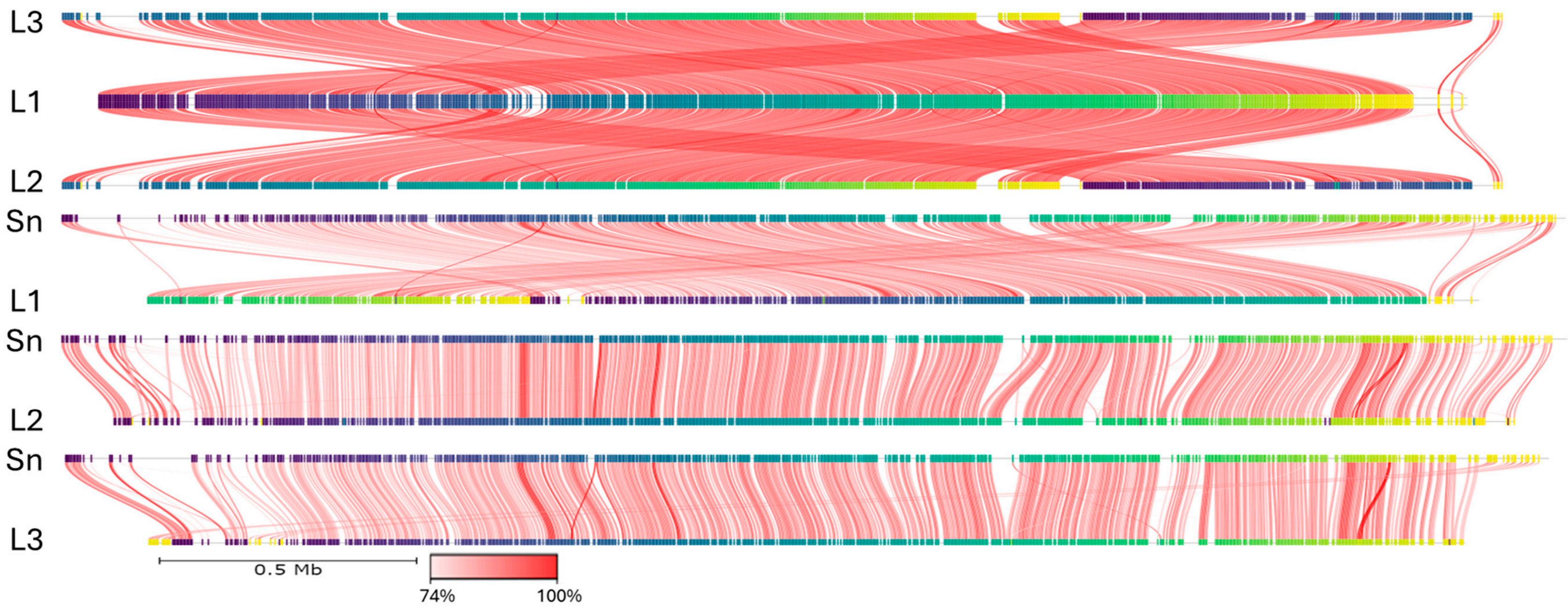

Figure S5. Graphical alignments of contigs containing Nat gene from three closely related *Staphylococcus cohnii* isolates from chickens. The upper panel is the graphical view of the Mauve alignment in MegAlignPro for the indicated contigs from isolates 1645, 1649, and 1701. For each contig sequence blocks are represented by colors. Below each contig graphic are the sequence ruler showing position in the contig, with the prokka annotation indicated by directional arrows. The bottom panel is an aligned histogram of SNP counts per 1000 bp in isolate 1645 relative to 1649 and 1701, based on the variants report for the orange sequence block region from MegAlignPro. The histogram shows significant variation for the first app. 23 kbp and the last app. 50 kbp of the aligned orange sequence block.

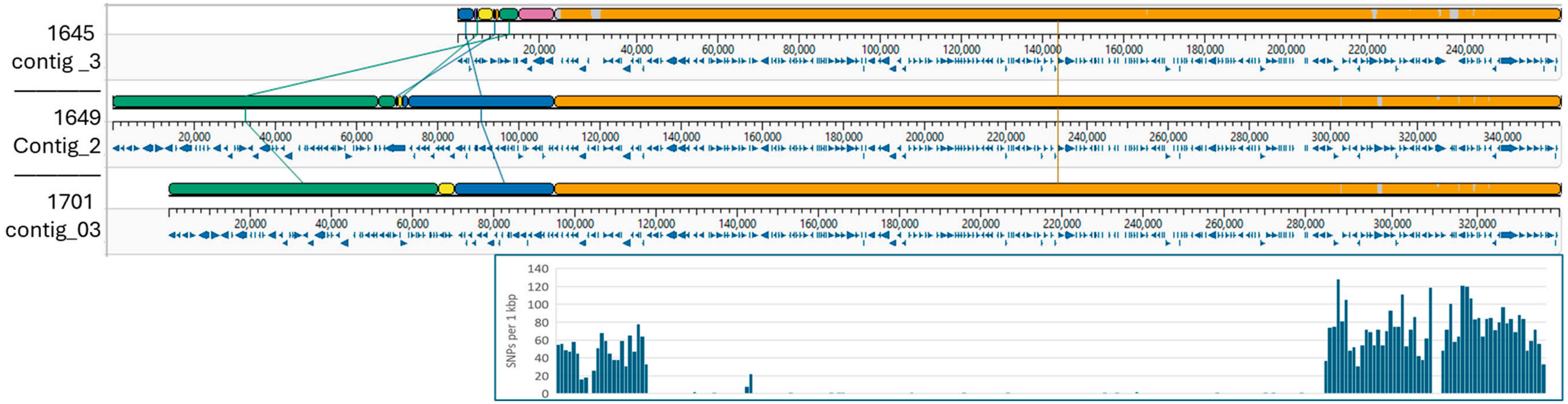

Figure S6. Clustal Omega alignments between *Staphylococcus cohnii* plasmids identified as sharing sequence identity in BLAST searches. Alignments were graphically depicted in MegAlignPro.

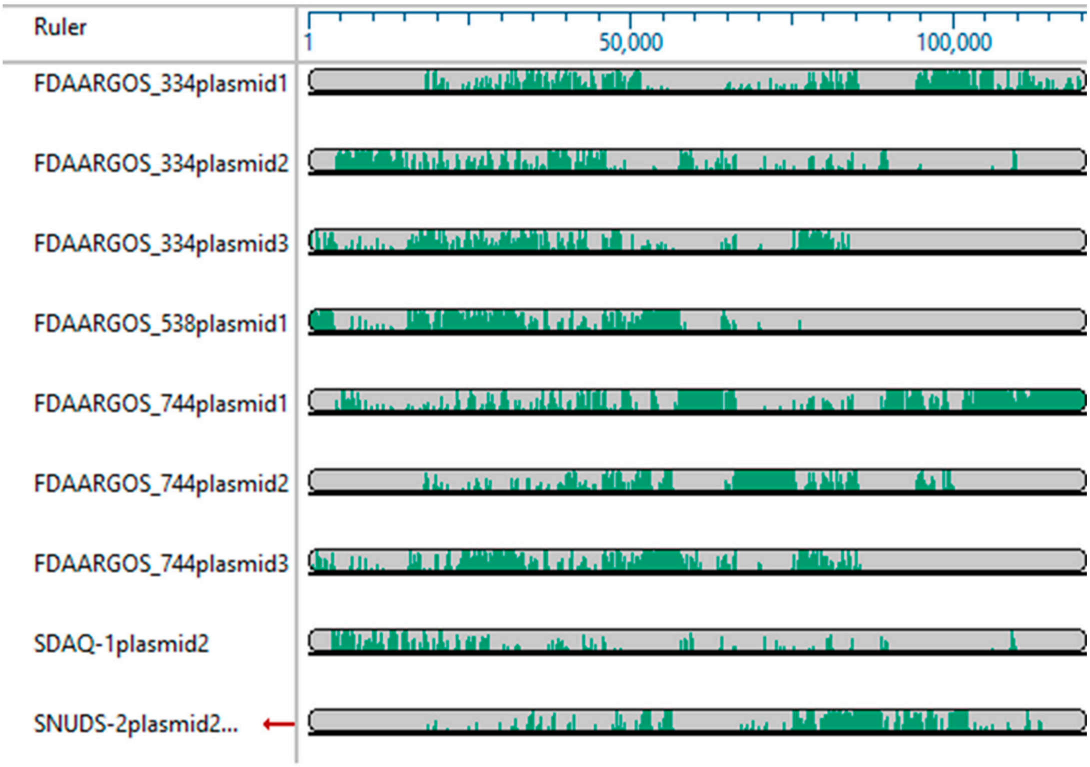

Supplement: Supplementary file 1 [file microorganisms-12-01549-s001.zip › Supplemental Figures.pdf]
